# A comparison of multiple imputation methods for handling missing values in longitudinal data in the presence of a time-varying covariate with a non-linear association with time: a simulation study

**DOI:** 10.1186/s12874-017-0372-y

**Published:** 2017-07-25

**Authors:** Anurika Priyanjali De Silva, Margarita Moreno-Betancur, Alysha Madhu De Livera, Katherine Jane Lee, Julie Anne Simpson

**Affiliations:** 1 Centre for Epidemiology and Biostatistics, Melbourne School of Population and Global Health, University of Melbourne, Melbourne, VIC Australia; 2 Clinical Epidemiology and Biostatistics Unit, Murdoch Childrens Research Institute, Royal Children’s Hospital, Melbourne, VIC Australia; 3 Department of Epidemiology and Preventive Medicine, Monash University, Melbourne, VIC Australia; 4 Department of Paediatrics, University of Melbourne, Melbourne, VIC Australia

**Keywords:** Fully conditional specification, Longitudinal data, Missing data, Multiple imputation, Multivariate normal imputation, Non-linear trajectory, Time-dependent covariate

## Abstract

**Background:**

Missing data is a common problem in epidemiological studies, and is particularly prominent in longitudinal data, which involve multiple waves of data collection. Traditional multiple imputation (MI) methods (fully conditional specification (FCS) and multivariate normal imputation (MVNI)) treat repeated measurements of the same time-dependent variable as just another ‘distinct’ variable for imputation and therefore do not make the most of the longitudinal structure of the data. Only a few studies have explored extensions to the standard approaches to account for the temporal structure of longitudinal data. One suggestion is the two-fold fully conditional specification (two-fold FCS) algorithm, which restricts the imputation of a time-dependent variable to time blocks where the imputation model includes measurements taken at the specified and adjacent times. To date, no study has investigated the performance of two-fold FCS and standard MI methods for handling missing data in a time-varying covariate with a non-linear trajectory over time – a commonly encountered scenario in epidemiological studies.

**Methods:**

We simulated 1000 datasets of 5000 individuals based on the Longitudinal Study of Australian Children (LSAC). Three missing data mechanisms: missing completely at random (MCAR), and a weak and a strong missing at random (MAR) scenarios were used to impose missingness on body mass index (BMI) for age z-scores; a continuous time-varying exposure variable with a non-linear trajectory over time. We evaluated the performance of FCS, MVNI, and two-fold FCS for handling up to 50% of missing data when assessing the association between childhood obesity and sleep problems.

**Results:**

The standard two-fold FCS produced slightly more biased and less precise estimates than FCS and MVNI. We observed slight improvements in bias and precision when using a time window width of two for the two-fold FCS algorithm compared to the standard width of one.

**Conclusion:**

We recommend the use of FCS or MVNI in a similar longitudinal setting, and when encountering convergence issues due to a large number of time points or variables with missing values, the two-fold FCS with exploration of a suitable time window.

**Electronic supplementary material:**

The online version of this article (doi:10.1186/s12874-017-0372-y) contains supplementary material, which is available to authorized users.

## Background

Epidemiological research has witnessed a major shift towards life-course studies which investigate how biological, behavioural, and physical exposures during gestation, childhood and adolescence are related to the development of disease in adulthood [1, 2]. Such studies involve following up individuals over a long period of time, with multiple waves of data collection, and consequently missing data are a major problem [3].

A number of statistical techniques have been developed to address missing data problems [4]. In the epidemiological literature, common approaches include complete case analyses and multiple imputation (MI) [5]. Another approach for longitudinal data is last observation carried forward; although this method has been shown to result in biased inference [6–8]. A complete case analysis, which only includes respondents with data available on all variables required for the target analysis, is commonly employed due to its simplicity. The validity of this approach relies on strong assumptions about the missing data, often requiring the stringent missing completely at random (MCAR) assumption, that there is no systematic difference between participants with complete and incomplete data [9]. An additional issue, particularly pertinent in longitudinal studies with several waves of data collection, is that a complete case analysis may include only a small and potentially unrepresentative sample of the original participants. MI was developed to address the limitations of a complete case analysis [10] and has grown in popularity over recent years [5]. MI is a two-stage process. In stage 1 the incomplete dataset is replicated multiple times and missing values are replaced by plausible values drawn from a posterior distribution according to a suitable imputation model based on the observed data. In stage 2 the target analysis is performed on each of the imputed datasets with the resulting parameter estimate and corresponding standard error of each dataset, combined into a single estimate (and standard error) [10]. The standard implementation of MI relies on the more relaxed missing at random (MAR) assumption, that the probability of a value missing is independent of unobserved data given the observed data [9]. MI enables all participants to be included in the analysis and may reduce bias and improve precision of the parameter estimates compared to a complete case analysis [9, 11].

Two standard MI methods have been proposed to impute missing data in the presence of multiple variables with missing values [5]. Multivariate normal imputation (MVNI) [12] fits a joint imputation model to all the variables containing missing data under the assumption that the variables follow a multivariate normal distribution [9]. Fully conditional specification (FCS), also known as multiple imputation by chained equations, fits separate univariate regression models to each variable with missing values [13–15], iteratively cycling through the univariate regression models. In longitudinal studies, missing data often occur in multiple variables across multiple waves. Both MVNI and FCS can be used to handle missing data in longitudinal studies by treating repeated measurements (i.e. same variable measured at different time points) as distinct variables in the imputation model (often referred to as “Just Another Variable”) [16]. However, this approach does not take into consideration the temporal trend in such variables across the waves [16–18]. Although such an approach may be adequate for a study with only a small number of time points (e.g. 3 waves of data collection) [19–22], when there are a large number of variables and time points, simulation studies (with 5 or more time points and/ or 3 or more variables with missing data) have shown that both MVNI and FCS in their standard form face convergence issues [17, 18]. This is primarily due to over-fitting of the imputation model and co-linearity between predictor variables [23]. This motivated the proposal of the two-fold FCS algorithm which imputes missing values at a certain time point based only on information from that time point and immediately adjacent time points [16]. The two-fold FCS method takes advantage of the temporal ordering of the repeated measurements to considerably reduce the number of predictor variables included in each of the univariate imputation models, and consequently diminishes over-fitting and co-linearity issues [17].

While there have been many studies evaluating MVNI and FCS methods to handle missing data in settings where the variables are measured at a single time point [9, 24–26], studies comparing MI methods in the context of longitudinal data are limited [18]. Welch et al. [17] performed a simulation study of 10 waves of data collection and 70% missing data, where explanatory variables were assigned to missing under a MCAR missing data mechanism. Although the study evaluated the performance of the standard FCS method and the two-fold FCS algorithm to handle missing data in time-dependent exposures, they included only time-independent and baseline values of the longitudinal variables as covariates in the target analysis [17]. This prevented them from observing how well the MI methods imputed missing values in the latter waves. A more comprehensive evaluation of MI methods for handling missing values in repeated measurements data was recently completed by Kalaycioglu et al. [18], comparing the performance of full Bayesian MI, MVNI, FCS and other variations of FCS for model reduction.

An important aspect that has not been explored in these studies is the performance of the various MI methods in the presence of a time-varying covariate with a non-linear association with time, a commonly encountered scenario in longitudinal observational studies [17, 27]. Not accounting for these non-linear trajectories in the imputation model (i.e. misspecification of imputation model) could potentially result in biased parameter estimates [28]. The aim of this paper was to assess the performance of MI methods in the context of an incomplete exposure with a non-linear association over time, considering methods that are available in standard statistical software (i.e. MVNI, FCS, and two-fold FCS) where up to 50% of data are MCAR or MAR. Specifically, we report the findings of a simulation study based on the Longitudinal Study of Australian Children (LSAC) [27], where there was interest in assessing the association between child’s body mass index (BMI) and sleep problems, both of which were measured repeatedly over five time points.

## Methods

### Motivating example: Longitudinal Study of Australian Children (LSAC)

*“Growing Up in Australia:* the Longitudinal Study of Australian Children (LSAC)” is a national longitudinal study of child and adolescent development. Two cohorts of children were recruited: the infant cohort (children born between March 2003 and February 2004) and the child cohort (born between March 1999 and February 2000). Wave 1 of data collection began in 2004 with subsequent waves every two years. Information was collected for each child on many areas including; children’s and parent’s physical and mental health, education, social and cultural environment, and family socio-economic position [27].

At the time of the current study we had access to five waves of data. Additional file 1: Table S1 provides details of waves 1 to 5 respondents.

### Epidemiological analysis of interest

Obesity is a common concern in Australian children [29], and may lead to a number of severe health problems in adulthood including cardiovascular disease, insulin resistance, and asthma [30, 31]. As sleep affects a child’s hormone related growth and maturation, it has a considerable impact on obesity [29, 32]. Conversely it has also been observed that childhood obesity could result in early life sleep problems such as obstructive sleep apnoea [33]. Therefore the relationship between increased rates of childhood obesity and childhood sleep problems is a research area that has gained much interest recently [29, 32, 33]. In LSAC, this question can be addressed by evaluating the association between BMI and sleep problems, and was the motivating example for the design of our simulation study.

#### Variables of interest

The exposure of interest was BMI for age z-score, which was measured repeatedly from ages 4 to 13 years. The raw BMI measurements were transformed into BMI for age z-scores (bmiz) as shown in Eq. 1 using the 2000 Centers for Disease Control (CDC) growth charts [27, 34]. The longitudinal outcome of interest was childhood sleep problems over the same time period, as reported by the primary care-giver (dichotomised into ‘no sleep problem’ (no or small) and ‘sleep problem’ (moderate or large) for our simulation study). Child’s sex, age, and weight at birth; maternal age at child birth, smoking, and education were considered as potential confounders [29, 35, 36]. Table 1 provides information on the variables used in the simulation study.

1 $$ bmiz=\frac{{\left(\frac{bmi}{M}\right)}^L-1}{SL}\  when\ L\ne 0;\kern0.5em  bmiz=\frac{\mathit{\ln}\left(\frac{bmi}{M}\right)}{S}\  when\ L=0 $$

where bmi corresponds to the raw BMI measurements, and the values for parameters L, M and S were obtained from the 2000 CDC growth charts based on the respondent’s age.

**Table 1:** Description of variables used in the simulation study, for the i^th^ child at wave j

Variable	Type	Grouping/ Units	Label
Study child’s BMI for age z-score	Continuous		bmiz_ij_
Study child’s sleep problems	Categorical	0 = No sleep problems 1 = Sleep problems	sleep_prob_ij_
Study child’s age	Continuous	Months	scage_ij_^a^
Maternal education	Categorical	0 = Not completed 1 = Completed	m_education_i_
Maternal smoking	Categorical	0 = No 1 = Yes	m_smoking_i_
Study child’s sex	Categorical	0 = Male 1 = Female	sex_i_
Study child’s birth weight	Continuous	Kilograms	birthweight_i_
Maternal age at child birth	Continuous	Years	m_age_i_

^a^A new variable scage_sq_ij_ was derived as the squared term of scage_ij_ to be used in the data generation models

#### Target analysis

The aim was to evaluate the population-average (marginal) association between bmiz (exposure) measured at one wave and sleep problems (outcome) measured at the subsequent wave based on the repeated measurements of sleep problems and bmiz. With complete data this parameter would be estimated using generalized estimating equations with a logit link and an unstructured correlation structure.

### Simulation of complete data

**Fig. 1:**
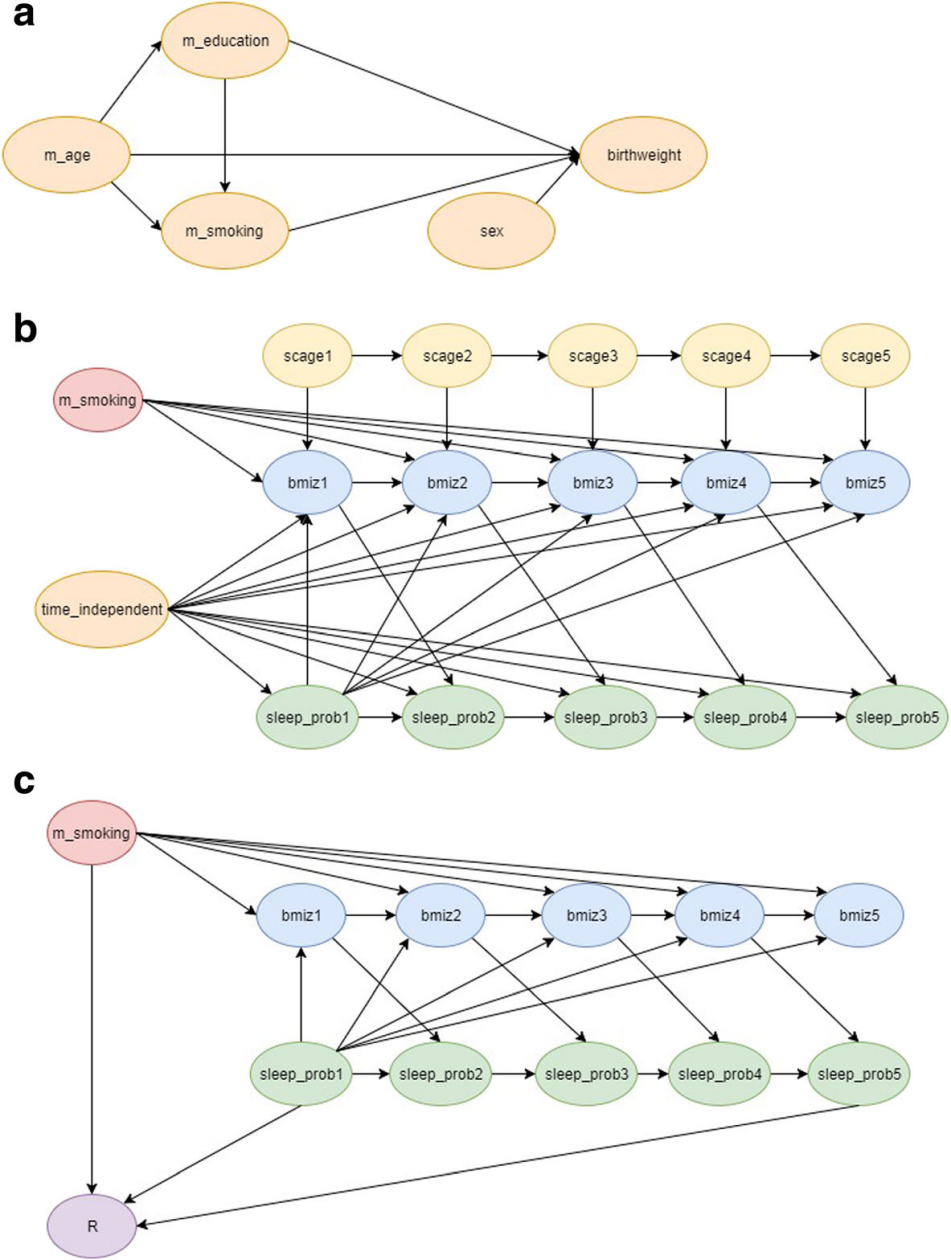
**a**) Variable dependencies of simulated time-independent variables; m_age, maternal age at child birth; m_education, maternal education; m_smoking, maternal smoking; sex, study child’s sex; birthweight, study child’s birth weight; **b**) Causal diagram for the association between sleep problems and BMI for age z-scores. For the ease of presentation all time-independent variables are presented using a single node excluding maternal smoking; scage1-scage5, study child’s age at waves 1 to 5; bmiz1-bmiz5, study child’s BMI for age z-scores at waves 1 to 5; sleep_prob1-sleep_prob5, study child’s sleep problems at waves 1 to 5; **c**) Causal diagram for MAR missingness. R is an indicator variable of missingness where BMI for age z-scores were assigned to missing if *R* = 1. Only variables required to model the MAR missingness are shown in the figure

Based on the child cohort of LSAC, which had a participation of 4983 children at wave 1 (Additional file 1: Table S1), we simulated 1000 datasets of 5000 individuals. Details of the simulation procedure are provided in the Additional file and variable labels used in the simulation equations are given in Table 1. Briefly, we first simulated the time-independent variables using the variable dependencies shown in Fig. 1a.

We then simulated the two time-dependent variables according to the following steps:

1. The sleep problem indicators at wave 1 were generated using the logistic regression model:

2 $$ \mathrm{logit}\ \left\{ \Pr \left( sleep\_{prob}_{i,1}=1\right)\right\}={\upeta}_0+{\upeta}_1\left[m\_{education}_i=1\right]+{\upeta}_2\left[{sex}_i=1\right]+{\upeta}_3{birthweight}_i+{\upeta}_4m\_{age}_i $$

2. For waves j = 1,…,5 bmiz measurements were generated using the linear mixed effects model:

3 $$ {bmiz}_{ij}=\left({\theta}_0+{a}_{0i}\right)+{\theta}_1 sleep\_{prob}_{i1}+\left({\theta}_2+{a}_{1i}\right){scage}_{ij}+\left({\theta}_3+{a}_{2i}\right){scage\_sq}_{ij}+{\theta}_4\left[m\_{education}_i=1\right]+{\theta}_5\left[m\_{smoking}_i=1\right]+{\theta}_6\left[{sex}_i=1\right]+{\theta}_7{birthweight}_i+{\theta}_8m\_{age}_i+{\varepsilon}_{ij} $$

where ε_ij_ is identically and independently distributed $\sim \mathrm{N}\ \left(0,{\upsigma}_{\upvarepsilon}^2\right)$. scage_sq_ij_ was derived as the squared term of scage_ij_ to incorporate the non-linear relationship between BMI for age z-scores with time. The random intercept a_0_, and random slopes a_1_ and a_2_ were drawn from a multivariate normal distribution. The mean and variance-covariance matrices, which were used to draw these random effects from a multivariate normal distribution, were obtained from the observed LSAC child cohort data.

3. The sleep problems indicators for waves j = 2,…,5 were then generated using the logistic regression model:

4 $$ \mathrm{logit}\ \left\{ \Pr \left( sleep\_{prob}_{i,j}=1\right)\right\}={\lambda}_0+{\lambda}_1{bmiz}_{i,j-1} + {\lambda}_2\left[m\_{education}_i=1\right]+{\lambda}_3\left[{sex}_i=1\right]+{\lambda}_4{birthweight}_i+{\lambda}_5m\_{age}_i + {\lambda}_6\left[ sleep\_{prob}_{i,j-1}=1\right] $$

The simulation model was designed to mimic the model for the epidemiological analysis of interest described previously so that λ_1_ of Eq. 4 is the true value for the parameter of interest. Parameter values used in the simulation process were chosen to mimic the LSAC data and are presented in Additional file 1: Table S2. We simulated data for sleep problems (waves j = 2,…,5) using odds ratios (ORs) that reflected scenarios of weak (OR = 1.1, λ_1_ = log(OR) = 0.1) and strong (OR = 1.5, λ_1_ = log(OR) = 0.4) associations between bmiz and sleep problems.

The casual diagram for the association between childhood sleep problems and bmiz is shown in Fig. 1b. Maternal smoking is presented as a separate node from the other time-independent variables as it is not in the analysis model but will be used in the imputation model as an auxiliary variable [11].

### Introduction of missing data

For each simulated dataset, bmiz measures were assigned to missing so by wave 5, 25% and 50% of these were missing (keeping bmiz at wave 1 as complete). Twenty-five percent was chosen to mimic the actual percentage of missing values in bmiz in LSAC child cohort (see Additional file 1: Figure S1) and 50% was chosen to represent a more extreme example, often observed in studies with long term follow-up [3]. The bmiz values at each wave were assigned to missing using either a MCAR scenario or one of the two MAR scenarios chosen to represent weak and strong associations between the indicator of missing bmiz (R) and the predictors of missingness. Under MCAR missingness, desired proportions of bmiz from waves 2–5 were randomly assigned to missing as intermittent missingness or missing for all subsequent waves (i.e. after a specific time point all bmiz measurements of a respondent are missing) as shown in Additional file 1: Figure S1.

Under MAR, it was assumed that the probability of missingness in bmiz at each wave followed a logistic regression model dependent on sleep problems measured at waves 1 and 5, and maternal smoking (Fig. 1c). Maternal smoking is not directly associated with the outcome and its effect on the outcome is only through bmiz (Fig. 1c). The presence of such a variable leads to bias in the complete case analysis [37].

Specifically, two logistic regression models were specified to generate missingness in bmiz from waves 2–5 under MAR, one to represent missing for all subsequent waves (Eq. 5) and the other to represent intermittent missingness (Eq. 6).

Model A: bmiz missing for all subsequent waves after wave j

5 $$ logit\left\{\mathit{\Pr}\left({R}_{i,j+1}=1\right)\right\}={\nu}_{0,j}+{\nu}_1\left[ sleep\_{prob}_{i,1}=1\right]+{\nu}_2\left[ sleep\_{prob}_{i,5}=1\right]+{\nu}_3\left[m\_{smoking}_i=1\right] $$

Model B: intermittent missingness between waves j and j + 1

6 $$ logit\left\{\mathit{\Pr}\left({R}_{i,j+1}=1\right)\right\}={\omega}_{0,j}+{\omega}_1\left[ sleep\_{prob}_{i,1}=1\right]+{\omega}_2\left[ sleep\_{prob}_{i,5}=1\right]+{\omega}_3\left[m\_{smoking}_i=1\right] $$

where model B was only applied to cases that were not specified as missing for all subsequent waves in model A. Assigned parameter values under the two MAR scenarios are given in Table 2.

**Table 2:** Specifications of the logistic regression models used to impose missing data under the MAR scenarios

Variable	Odds Ratio			
	MAR (weak)		MAR (strong)^a^	
	Model A/ Eq. 5 ^b^ (exp(*ν*_*i*_))	Model B/ Eq. 6 ^b^ (exp(*ω*_*i*_))	Model A/ Eq. 5 ^b^ (exp(*ν*_*i*_))	Model B/ Eq. 6 ^b^ (exp(*ω*_*i*_))
1 Sleep problem at wave 1_Yes_	1.67	1.61	2.80	2.60
2 Sleep problem at wave 5_Yes_	1.64	1.58	2.70	2.50
3 Maternal smoking_Yes_	1.61	1.58	2.60	2.50

*exp* exponential, *MAR* missing at random

^a^Odds ratio for MAR (Strong) = square of the Odds ratio for MAR (Weak)

^b^Model A/ Eq. 5 and Model B/ Eq. 6 represent the logistic regression models used to generate missingness in BMI for age z-scores from waves 2–5 under MAR, to denote bmiz missing for all subsequent waves and intermittent missingness respectively

The intercepts for the logistic regression models, *ν*_0 , *j*_ (Eq. 5) and *ω*_0 , *j*_ (Eq. 6), were chosen by iteration to achieve the required proportions of intermittent missingness or missing for all subsequent waves in bmiz at each wave. For the strong MAR scenario, we doubled the log of the ORs used in the weak MAR scenario.

### Methods to handle missing data

We compared the performance of three MI methods; MVNI, FCS and two-fold FCS and additionally conducted a complete case analysis as it is a commonly used approach to handle missing data [5, 28]. For the complete case analysis all individuals with any missing values in bmiz measured at waves 2 to 4 were excluded from the analysis. Under the three MI methods, the imputation model included all variables in the analysis model, and the auxiliary variable, maternal smoking, and 50 imputations were performed. Standard implementation of MVNI and FCS (Stata commands ‘mi impute mvn’ and ‘mi impute chained’ respectively) handled the repeated measures of bmiz and sleep problems by including the repeated measurements as distinct variables in the imputation model. In the two-fold FCS method (Stata command ‘twofold’), the longitudinal structure of the variables was taken into consideration and missing values were imputed using information at the specified and immediately adjacent time points.

### Performance measures for evaluating different methods

The target analysis parameter of interest was the log(OR) for the association between sleep problems and bmiz measured at a previous wave, estimated using generalized estimating equations to account for repeated measures (see Epidemiological Analysis section). The true value of the parameter of interest was the value used in the simulation model for the outcome variable sleep problems in Eq. 4 (log(1.1) and log(1.5)). For each of the different simulation scenarios, the performances of the complete case analysis and the three MI methods were evaluated using the absolute bias, defined as the difference between the true value and average of parameter estimates calculated across the 1000 simulated datasets; the empirical standard error, calculated as the square root of the variance of the estimates across the 1000 datasets; and coverage probability of the 95% confidence interval, estimated by the proportion of datasets where the estimated 95% confidence interval contained the true parameter value. We also reported the relative bias, defined as the bias relative to the true value, the model-based standard error (i.e. the arithmetic mean of standard errors across the 1000 simulated datasets) and the root mean square error, which is computed as a combination of the bias and variance of the estimate [38]. The Monte Carlo error for the MI estimate was also extracted, which describes how an estimated statistic deviates over repeated simulations [39]. An acceptably small Monte Carlo error in the MI estimate would be expected when the number of imputations is equal to the percentage of individuals with missing values (i.e. 25 imputations are used when the percentage of respondents with missing data is approximately 25%) [4].

All data simulation and analyses were conducted using Stata version 13.1 [40].

## Results

**Fig. 2:**
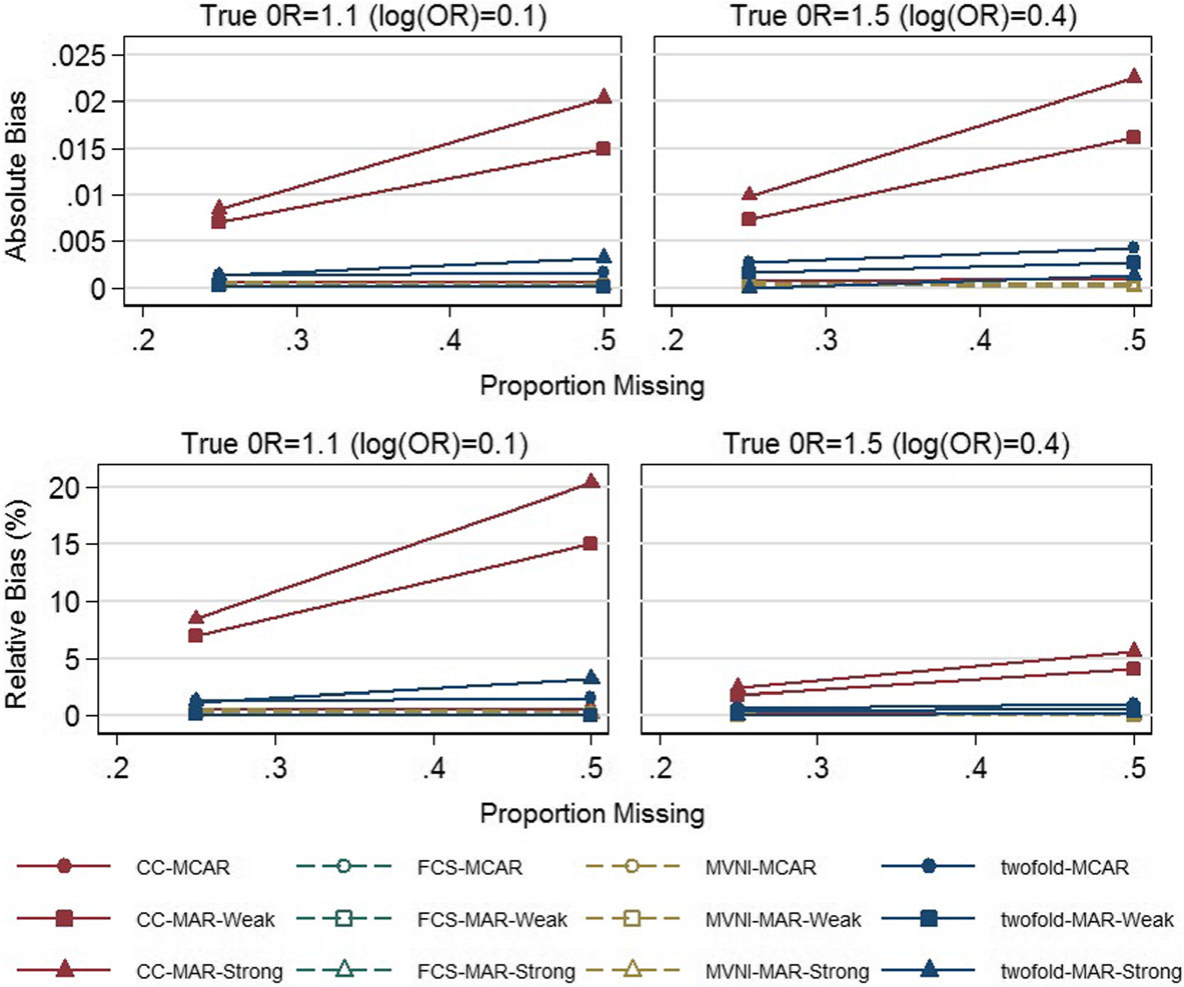
Absolute bias and Relative bias (%) for complete case analysis (CC), fully conditional specification (FCS), multivariate normal imputation (MVNI), and two-fold fully conditional specification (two-fold FCS) for increasing proportions of missing data (0.25, 0.5) under three missing data scenarios and two simulation scenarios; true OR represents the true odds ratio between sleep problems and BMI for age z-scores. ^a^Relative bias is calculated as absolute bias relative to the value of the true parameter. As the value of the true parameter (log(OR)) increases from 0.1 to 0.4 in the second simulation scenario, the magnitude of the relative bias drops even though the absolute bias shows a slight increase

Table 3, Additional file 1: Tables S3, S4 and S5 summarize the performance of three MI methods; FCS, MVNI and two-fold FCS, and complete case analysis, across the different simulation scenarios described above. We observed minimal bias in the presence of 25% missing data under all missing data scenarios (MCAR, MAR (weak), and MAR (strong)) and all missing data methods, with the bias not exceeding 0.02. Increasing the proportion of missing bmiz values to 50%, we observed moderate bias when using complete case analysis under the two MAR missing data scenarios (Fig. 2; relative bias ranged from 4% to 20%). We observed minimal bias for both FCS and MVNI, while the two-fold FCS produced a slightly higher level of bias, albeit minimal (relative bias ranged from 0.002% to 3%).

**Table 3:** Performance of various methods for handling 50% missingness in BMI for age z-scores; true OR^a^ = 1.1(log(OR) = 0.1)

Performance Measure	Method				
	Complete Case Analysis	FCS	MVNI	two-fold FCS (width = 1)^c^	two-fold FCS (width = 2)^d^
MCAR					
Absolute Bias^b^	0.001	0.000	0.000	0.002	0.001
Relative Bias (%)	0.65	0.28	0.34	1.63	0.77
Empirical SE	0.017	0.017	0.017	0.018	0.017
Model-based SE	0.018	0.017	0.017	0.017	0.017
Coverage (%)	95.6	95.8	95.9	95.3	95.9
RMSE	0.017	0.017	0.017	0.018	0.017
MAR (weak)					
Absolute Bias^b^	0.015	0.000	0.000	0.000	0.000
Relative Bias (%)	15.03	0.16	0.22	0.03	0.28
Empirical SE	0.018	0.017	0.017	0.017	0.017
Model-based SE	0.018	0.017	0.017	0.017	0.017
Coverage (%)	86.3	94.4	94.5	94.3	94.6
RMSE	0.023	0.017	0.017	0.017	0.017
MAR (strong)					
Absolute Bias^b^	0.020	0.000	0.000	0.003	0.002
Relative Bias (%)	20.36	0.23	0.21	3.19	2.16
Empirical SE	0.018	0.017	0.017	0.018	0.017
Model-based SE	0.017	0.017	0.017	0.017	0.017
Coverage (%)	77.8	95.0	94.9	93.6	93.9
RMSE	0.027	0.017	0.017	0.018	0.018

*Empirical SE* empirical standard error, *FCS* fully conditional specification, *MAR* missing at random, *MCAR* missing completely at random, *Model-based SE* model based standard error, *MVNI* multivariate normal imputation, *RMSE* root mean square error, *two-fold FCS* two-fold fully conditional specification algorithm

^a^True OR represents the true odds ratio between sleep problems and BMI for age z-scores

^b^Monte Carlo standard error did not exceed 0.0006

^c^Results for the two-fold FCS with a time window width of 1, that is, including immediately adjacent time points

^d^Results for the two-fold FCS with a time window width of 2, that is, including two adjacent time points

**Fig. 3:**
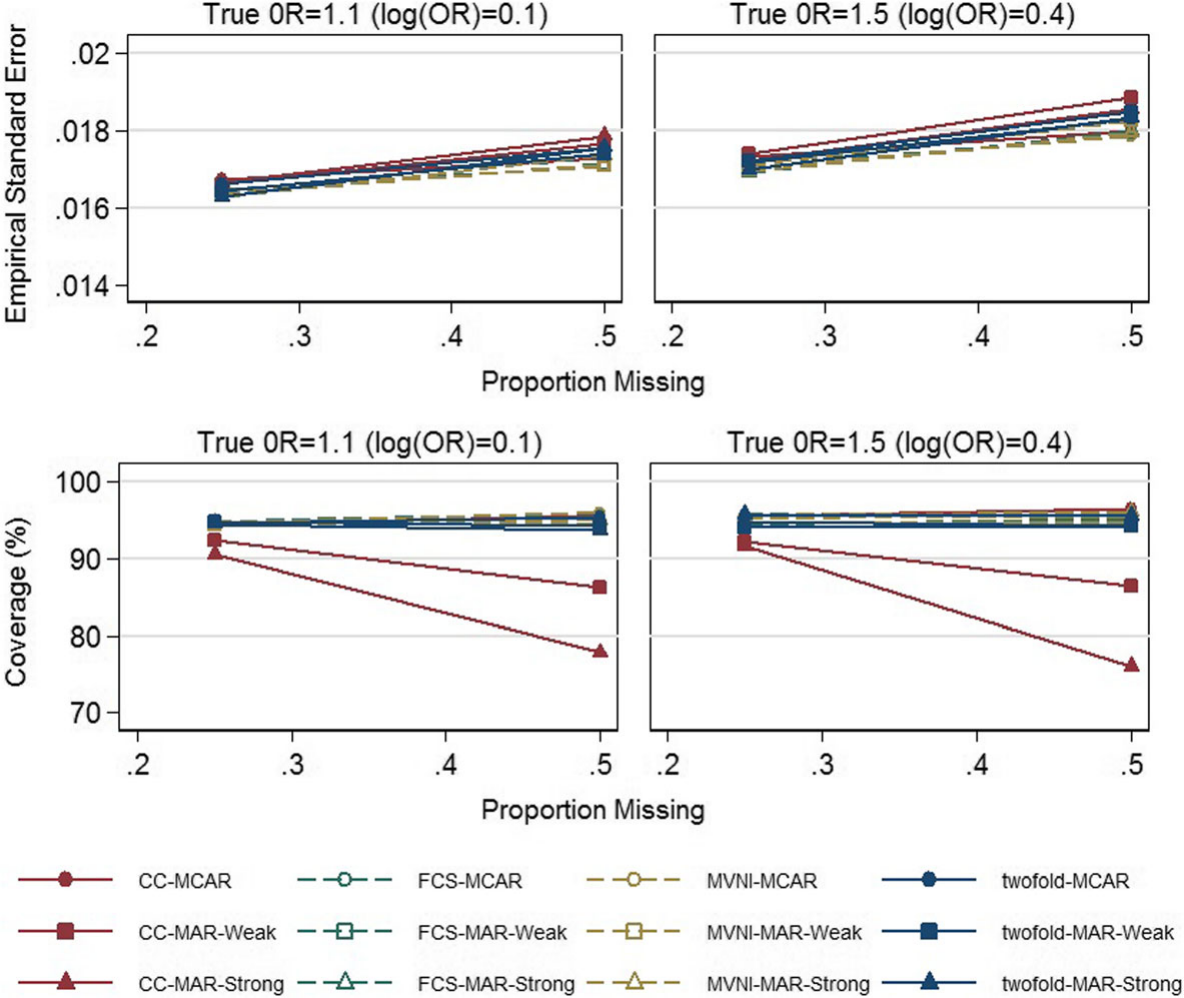
Empirical standard error and Coverage (%) for complete case analysis (CC), fully conditional specification (FCS), multivariate normal imputation (MVNI), and two-fold fully conditional specification (two-fold FCS) for increasing proportions of missing data (0.25, 0.5) under three missing data scenarios and two simulation scenarios; true OR represents the true odds ratio between sleep problems and BMI for age z-scores

The empirical standard errors are shown in Fig. 3. We observed similar empirical standard errors for all approaches. The root mean square error increased with the proportion of missingness, and for MAR compared with MCAR, and MI methods showed a gain in root mean square error under the strong MAR scenario with 50% missing data (see Additional file 1: Figure S2).

As expected the coverage remained within 93.6% and 96.4% for the nominal level of 95% for all scenarios (based on number of simulations) except when using complete case analysis under the weak and strong MAR scenarios, which reported a slight undercoverage (Fig. 3).

Additional file 1: Figures S3 and S4 compare the performance of the two-fold FCS algorithm with a time window width of 1 and 2. As expected, we observed slight improvements in bias and precision when using a time window width of 2, compared to the standard two-fold FCS algorithm, as more information is being used to impute the missing values.

## Discussion

We evaluated the performance of MI methods, MVNI, FCS and two-fold FCS, and complete case analysis for handling up to 50% missing data in a longitudinal exposure variable which had a non-linear association with time, using a simulation study designed based on the LSAC child cohort. We found very little bias and coverage remained around 95% for the three MI methods; MVNI, FCS and two-fold FCS (using a time window width of 1 and 2), whereas moderate bias was observed for complete case analysis when there was 50% MAR missing data. We also observed slight gains in precision from all MI methods when compared with a complete case analysis. The two-fold FCS produced slightly more biased and less precise estimates than FCS and MVNI when using adjacent time points only, however, these differences were minimal. The simulations didn’t reveal too large biases for any of the MI approaches in any of the scenarios. Our results reflect what may actually be expected in practice as we have assessed realistic scenarios by basing our simulations on the LSAC study.

Our findings are consistent with the results of a simulation study conducted by Kalaycioglu et al. [18], which focused on a continuous longitudinal outcome, showing that MI provided greater precision compared with complete case analysis especially when the outcome variable was fully observed.

We used maternal smoking measured at baseline as an auxiliary variable. In the statistical literature it has been observed that if the imputation model contains auxiliary variables with strong associations with the variable subject to missingness, MI could result in slight gains in precision compared with a complete case analysis [41, 42].

Also similar to findings of past literature, we observed hardly any difference in bias and precision between FCS and MVNI [24]. Kalayciolgu et al. [18] recommended MVNI over FCS approaches when imputing longitudinal continuous exposures as it assumes an unstructured correlation structure, which is more flexible for repeated measurements data, while FCS approaches are more suitable for repeated measurements with an auto-regressive correlation structure.

Our observations differed slightly from the findings of Welch et al. [17], another simulation study comparing standard and two-fold FCS methods. Welch et al. reported unbiased and more precise estimates using two-fold FCS compared to standard FCS, with the latter failing to converge in approximately 25% of the simulated datasets due to potential co-linearity issues. While the study by Welch et al. [17] had 10 time points and many variables subject to missingness, we used 5 time points and one variable with missing values in our simulation study. Past literature shows that FCS fails to converge more readily when imputing many longitudinal variables subject to missingness [18]. Of note, the target analysis model used by Welch et al. only took into consideration the baseline values of the longitudinal variables restricting their evaluation of how the MI methods imputed missing values in latter waves [17]. By using a generalized estimating equation as the analysis model we were able to use information from all time points to estimate the parameter of interest, enabling us to conduct a more comprehensive evaluation of how the MI methods handled missing values in repeated measurements. Our findings of slightly more bias and less precision when using the two-fold FCS compared to FCS and MVNI may be due to the continuous time-varying exposure variable with missing data having a non-linear trajectory over time. As the two-fold FCS uses information only from the specified and immediately adjacent time points to impute missing values, the non-linear trajectory over time might not be captured sufficiently, potentially resulting in biased estimates with less precision compared to FCS and MVNI [17]. When we increased the width of the two-fold FCS algorithm to include two adjacent time points we observed slightly less biased and more precise estimates, implying that the non-linear trajectory over time could be captured better by increasing the time window width.

The structure of longitudinal studies is becoming more complex, with studies often including a large number of time points and having an unbalanced design [43]. If standard FCS and MVNI are more likely to fail, and if the two-fold FCS algorithm potentially introduces bias if a large enough time window width is not considered, alternative methods to handle missing data might be required. Direct likelihood analysis based on a generalized linear mixed-effects model and the ‘jomo’ package in R [44] are alternative approaches to handle missing values in longitudinal data. The generalized linear mixed-effects model is suitable for handling missing values in a time-dependent outcome variable as it allows the inclusion of all respondents in the analytical process, given that they have at least one outcome measure, and it captures the longitudinal structure of the data [45]. Unlike in MI, it does not suffer from incompatibility issues between the target analysis model and imputation models as only one model is specified within this approach, and any non-linear associations and/ or interactions are directly incorporated into the model. ‘jomo’ is a package specifically for multilevel joint modelling MI, which can handle both incomplete covariates and outcomes. Within this package, cluster-specific covariance matrices can be used for imputation of missing values in clustered data [44]. While the package is not yet widely adopted, it was only recently extended for handling missing values in repeated measurements, and has only been evaluated for a small number of time points [44].

Simulation studies based on real cohort studies have been frequently used in the statistical literature [41, 46–51]. Using an existing cohort study allowed us to incorporate complex yet realistic associations into the simulated data. We evaluated varied percentages of missing data, different missing data mechanisms, and varied levels of dependencies in the predictors of missing data. The generalizability of our results is limited since the simulation study was designed based on a single cohort. Therefore it would be useful to further explore other simulation models based on real data scenarios to provide more evidence regarding the performance of the MI methods [41].

The MI methods evaluated in our study require the MAR assumption to produce unbiased estimates [9]. However missing data could also be missing not at random, which is when missingness is dependent on both observed and missing data [52]. Further research on sensitivity analysis methods to assess deviations from MAR in the longitudinal setting is important [5, 53], however, was beyond the scope of our paper.

## Conclusion

The findings from this simulation study, which was designed based on a longitudinal cohort study, indicate that FCS and MVNI perform better than the two-fold FCS in terms of bias and precision, when handling up to 50% missing values in a time-varying covariate with a non-linear trajectory over time. In a similar longitudinal setting we would generally recommend the use of MVNI or FCS, instead of the two-fold FCS algorithm. However, if faced with convergence issues due to a large number of time points or variables with missing values, the two-fold FCS algorithm would be an appropriate method to use providing that a suitable time window is used in the imputation model. Of course, caution is required as these recommendations are based on a single simulation study and further research is warranted.

## Additional file

Additional file 1: Supplementary Material. (DOCX 550 kb)

## Abbreviations

BMI: Body mass index
bmiz: BMI for age z-scores
FCS: Fully conditional specification
LSAC: Longitudinal study of Australian children
MAR: Missing at random
MCAR: Missing completely at random
MI: Multiple imputation
MVNI: Multivariate normal imputation
OR: Odds ratio
two-fold FCS: two-fold fully conditional specification
