# Supplementary material for: A comparison of multiple imputation methods for handling missing values in longitudinal data in the presence of a time-varying covariate with a non-linear association with time: a simulation study
Source: BMC Med Res Methodol. 2017 Jul 25;17:114. doi: 10.1186/s12874-017-0372-y (PMC5526258; doi:10.1186/s12874-017-0372-y)
Supplement: Supplementary file 1 — Supplementary Material. (DOCX 550 kb) [file 12874_2017_372_MOESM1_ESM.docx]

**Additional files**

Table S1. LSAC respondents by wave of data collection

| Infant cohort | Wave 1  2004 | Wave 2  2006 | Wave 3  2008 | Wave 4  2010 | Wave 5  2012 |
| --- | --- | --- | --- | --- | --- |
| Age (years) | 0 – 1 | 2 – 3 | 4 -5 | 6 - 7 | 8 - 9 |
| Number of participants | 5107 | 4606 | 4386 | 4242 | 4085 |
| Unit non-response |  | 10% | 14% | 17% | 20% |
| Child cohort | Wave 1  2004 | Wave 2  2006 | Wave 3  2008 | Wave 4  2010 | Wave 5  2012 |
| Age (years) | 4 – 5 | 6 – 7 | 8 – 9 | 10 – 11 | 12 - 13 |
| Number of participants | 4983 | 4464 | 4331 | 4169 | 3956 |
| Unit non-response |  | 10% | 13% | 16% | 21% |

**Simulation of complete data**

The simulation study was based on the child cohort of the Longitudinal Study of Australian Children (LSAC), which had a participation of 4983 children in its first wave of data collection (Supplementary Table 1). Data were generated as specified below for each child i= 1,..., *N*, where N = 5000 for waves j=1,…,5. A total of 1000 datasets were simulated.

Parameters used in the simulation process were chosen to mimic the LSAC data and are presented in Supplementary Table 2. Refer to Table 1 in the main text for information on the variable labels used in the simulation equations specified below.

1. Generated maternal age at child birth (m_age_i_) from a normal distribution.
2. Generated maternal education (m_education_i_) as a time independent variable, from a logistic regression model (Equation S1) conditional on maternal age at child birth.

$logit \left\{ \Pr\left( {m\_education}_{i}=1 \right) \right\}= \pi_{0}+ \pi_{1}{m\_age}_{i}$ (S1)

1. Generated maternal smoking (m_smoking_i_) as a time independent variable, from a logistic regression model (Equation S2) conditional on maternal age at child birth and maternal education at baseline.

$logit \left\{ \Pr\left( {m\_smoking}_{i}=1 \right) \right\}= \tau_{0}+ \tau_{1}{m\_age}_{i} + \tau_{2}[{m\_education}_{i}=1]$ (S2)

1. Generated study child’s sex (sex_i_) by randomly assigning a p% of respondents to be female.
2. Generated study child’s birth weight (birthweight_i_) from a linear regression model (Equation S3) conditional on maternal age at child birth, maternal education, maternal smoking and study child’s sex.

$${birthweight}_{i}= \gamma_{0}+ \gamma_{1}{m\_age}_{i}+ \gamma_{2}\left[ {m\_education}_{i}=1 \right]+ \gamma_{3}\left[ {m\_smoking}_{i}=1 \right]+ \gamma_{4}\left[ {sex}_{i}=1 \right]+ \varphi_{i}$$

(S3)

Where $\varphi_{i}$ is identically and independently distributed as; $\varphi_{i}\sim N (0, \sigma_{\varphi}^{2})$

1. Generated study child’s age for wave 1 (scage_i1_) from a normal distribution as; ${scage}_{i1} \sim N (57, {2.6}^{2})$. The study child’s age for the succeeding waves was generated by adding the time gap between the waves to the age of the preceding wave (24 months) (Equation S4).

${scage}_{ij}= 24+ {scage}_{ij-1}$ (S4)

1. Generated time dependent variables; study child’s sleep problems (sleep_prob_ij_) and BMI for age z-scores (bmiz_ij_), for waves 1 to 5 as mentioned in the main text, using following steps;

- Generated sleep problems at wave 1 using a logistic regression model (Equation S5).

$$\mathrm{logit}\left\{ \Pr\left( {sleep\_prob}_{i,1}=1 \right) \right\}= \eta_{0}+ \eta_{1}\left[ {m\_education}_{i}=1 \right]+\eta_{2}\left[ {sex}_{i}=1 \right]+ \eta_{3}{birthweight}_{i}+ \eta_{4}{m\_age}_{i}$$

(S5)

- Generated bmiz for waves j=1,…,5 using a linear mixed effects model (Equation S6).

${bmiz}_{ij}=\left( \theta_{0}+a_{0i} \right)+{{\theta_{1}{sleep\_prob}_{i1}+(\theta}_{2}+a_{1i}){scage}_{ij}+{(\theta}_{3}{+a_{2i})scage\_sq}_{ij}+ \theta}_{4}\left[ {m\_education}_{i}=1 \right]+ \theta_{5}\left[ {m\_smoking}_{i}=1 \right]+ \theta_{6}\left[ {sex}_{i}=1 \right]+\theta_{7}{birthweight}_{i}+ \theta_{8}{m\_age}_{i}{+ \varepsilon}_{ij}$ (S6)

where $\varepsilon_{\mathrm{ij}}$ is identically and independently distributed $\sim N (0, \sigma_{\varepsilon}^{2})$ . scage_sq_ij_ was derived as the squared term of scage_ij_ to incorporate the non-linear relationship between BMI for age z-scores with time. The random intercept a_0_, and random slopes a_1_ and a_2_ were drawn from a multivariate normal distribution. The mean and variance-covariance matrices, which were used to draw these random effects from a multivariate normal distribution, were obtained from the observed LSAC child cohort data.

- Sleep problems for wave j=2,…,5 were then generated using a logistic regression model as mentioned in the main text (Equation S7). The simulation model was designed to mimic the model for the epidemiological analysis, so that the true parameter value is known.

$\mathrm{logit}\left\{ \Pr\left( {sleep\_prob}_{i,j}=1 \right) \right\}= \lambda_{0}+ \lambda_{1}{bmiz}_{i,j-1} {+ \lambda}_{2}{[m\_education}_{i}=1]+\lambda_{3}{[sex}_{i}=1]+\lambda_{4}{birthweight}_{i}+\lambda_{5}{m\_age}_{i} +\lambda_{6}{[sleep\_prob}_{i,j-1}=1]$ (S7)

Table S2: Details of the parameters used in the data generation models

| Variable Generated |  | Explanatory Variable | | Parameter Value |
| --- | --- | --- | --- | --- |
| Maternal age at child birth (years) |  |  | Mean | 30 |
|  |  |  | Standard deviation | 5 |
|  |  |  |  |  |
| Maternal education |  | Constant | $\pi_{0}$ | -1.3 |
|  |  | Maternal age, years | $\pi_{1}$ | 0.1 |
|  |  |  |  |  |
| Maternal smoking |  | Constant | $\tau_{0}$ | 2.5 |
|  |  | Maternal age, years | $\tau_{1}$ | -0.1 |
|  |  | Maternal education_Completed_ | $\tau_{2}$ | -0.6 |
|  |  |  |  |  |
| Study child’s sex |  | Female proportion | p | 0.5 |
|  |  |  |  |  |
| Study child’s birth weight (kg) |  | Constant | $\gamma_{0}$ | 3.4 |
|  |  | Maternal age, years | $\gamma_{1}$ | 0.01 |
|  |  | Maternal education_Completed_ | $\gamma_{2}$ | 0.05 |
|  |  | Maternal smoking_Yes_ | $\gamma_{3}$ | -0.1 |
|  |  | Study child’s sex_Female_ | $\gamma_{4}$ | -0.1 |
|  |  | Error term | Mean | 0 |
|  |  |  | Standard deviation $\sigma_{\varphi}$ | 0.6 |
|  |  |  |  |  |
| Study child’s age at wave 1 (months) |  |  | Mean | 57 |
|  |  |  | Standard deviation | 2.6 |
|  |  |  |  |  |
| Sleep problems at wave 1 |  | Constant | $\eta_{0}$ | 1.1 |
|  |  | Maternal education_Completed_ | $\eta_{1}$ | -0.2 |
|  |  | Study child’s sex_Female_ | $\eta_{2}$ | -0.1 |
|  |  | Birth weight, kg | $\eta_{3}$ | -0.2 |
|  |  | Maternal age, years | $\eta_{4}$ | 0.01 |
|  |  |  |  |  |
| BMI for age z-scores |  | Constant | $\theta_{0}$ | -0.1 |
|  |  | Sleep problem at wave 1_Yes_ | $\theta_{1}$ | 0.5 |
|  |  | Study child’s age, months | $\theta_{2}$ | -0.001 |
|  |  | Study child’s age squared | $\theta_{3}$ | -0.00005 |
|  |  | Maternal education_Completed_ | $\theta_{4}$ | -0.1 |
|  |  | Maternal smoking_Yes_ | $\theta_{5}$ | 0.5 |
|  |  | Study child’s sex_Female_ | $\theta_{6}$ | -0.002 |
|  |  | Birth weight, kg | $\theta_{7}$ | 0.3 |
|  |  | Maternal age, years | $\theta_{8}$ | 0.001 |
|  |  | Error term | Mean | 0 |
|  |  |  | Standard deviation $\sigma_{\varepsilon}$ | 0.6 |
|  |  | Random Effects   - Standard deviation - Correlations | b_0_  b_1_  b_2_  b_0_ b_1_  b_0_ b_2_  b_1_ b_2_ | 1.8  0.03  0.0001  -0.9  0.8  -0.97 |
|  |  |  |  |  |
| Sleep problems measured at waves j=2,…,5 |  | Constant | $\lambda_{0}$ | -1.2 |
|  |  | bmiz at previous wave | $\lambda_{1}$ | 0.1 / 0.4^a^ |
|  |  | Maternal education_Completed_ | $\lambda_{2}$ | -0.6 |
|  |  | Study child’s sex_Female_ | $\lambda_{3}$ | -0.4 |
|  |  | Birth weight, kg  Maternal age, years  Sleep problem at previous wave_Yes_ | $\lambda_{4}$  $\lambda_{5}$  $\lambda_{6}$ | -0.5  0.1  1.7 |
|  |  | | | |

Abbreviations: bmiz, BMI for age z-scores; kg, kilograms.

^a^ We simulated data for sleep problems for waves j=2,…,5 using two odds ratios (ORs) 1.1 (log(OR)=0.1) and 1.5 (log(OR)=0.4) to represent a weak and strong association between bmiz measured at a previous wave and sleep problems measured at a later wave.


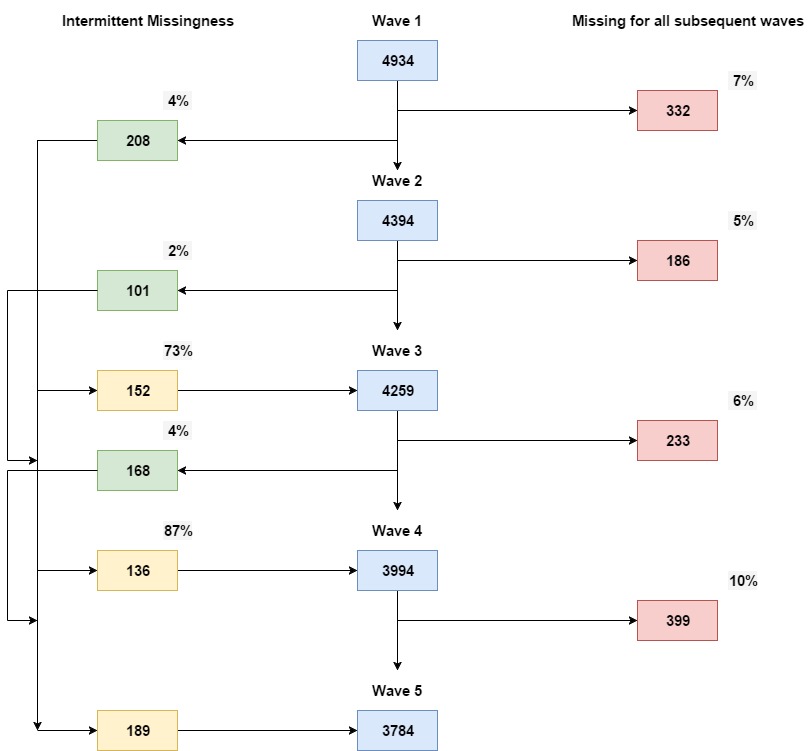


Figure S1. Missing data patterns for BMI for age z-scores (bmiz) in the original child cohort of the Longitudinal Study of Australian Children. Percentage of respondents who had all bmiz measurements missing after each wave is presented using missing for all subsequent waves, and who had bmiz measured at a later wave are presented as intermittent missingness.

Table S3. Performance of various methods for handling 25% missingness in BMI for age z-scores; true OR^a^=1.1(log(OR)=0.1)

| Performance Measure | Method | | | | |
| --- | --- | --- | --- | --- | --- |
|  | Complete Case Analysis | FCS | MVNI | two-fold FCS (width=1)^c^ | two-fold FCS (width=2)^d^ |
| **MCAR** |  |  |  |  |  |
| Absolute Bias^b^ | 0.001 | 0.001 | 0.001 | 0.001 | 0.001 |
| Relative Bias (%) | 0.54 | 0.56 | 0.54 | 1.26 | 0.57 |
| Empirical SE^b^ | 0.017 | 0.016 | 0.016 | 0.017 | 0.017 |
| Model-based SE^b^ | 0.016 | 0.016 | 0.016 | 0.016 | 0.016 |
| Coverage (%) | 94.5 | 94.8 | 94.6 | 94.6 | 94.4 |
| RMSE | 0.017 | 0.016 | 0.016 | 0.017 | 0.017 |
| **MAR (weak)** |  |  |  |  |  |
| Absolute Bias^b^ | 0.007 | 0.000 | 0.000 | 0.000 | 0.000 |
| Relative Bias (%) | 7.02 | 0.24 | 0.26 | 0.23 | 0.09 |
| Empirical SE^b^ | 0.017 | 0.016 | 0.016 | 0.016 | 0.016 |
| Model-based SE^b^ | 0.016 | 0.016 | 0.016 | 0.016 | 0.016 |
| Coverage (%) | 92.4 | 94.5 | 94.4 | 94.7 | 94.7 |
| RMSE | 0.018 | 0.016 | 0.016 | 0.016 | 0.016 |
| **MAR (strong)** |  |  |  |  |  |
| Absolute Bias^b^ | 0.008 | 0.000 | 0.000 | 0.001 | 0.001 |
| Relative Bias (%) | 8.44 | 0.27 | 0.30 | 1.26 | 0.87 |
| Empirical SE^b^ | 0.017 | 0.016 | 0.016 | 0.016 | 0.016 |
| Model-based SE^b^ | 0.016 | 0.016 | 0.016 | 0.016 | 0.016 |
| Coverage (%) | 90.6 | 94.7 | 94.7 | 94.5 | 94.6 |
| RMSE | 0.019 | 0.016 | 0.016 | 0.016 | 0.016 |

Abbreviations: Empirical SE, empirical standard error; FCS, fully conditional specification; MAR, missing at random; MCAR, missing completely at random; Model-based SE, model based standard error; MVNI, multivariate normal imputation; RMSE, root mean square error; two-fold FCS, two-fold fully conditional specification algorithm.

^a^True OR represents the true odds ratio between sleep problems and BMI for age z-scores.

^b^Monte Carlo standard error did not exceed 0.0006.

^c^Results for the two-fold FCS with a time window width of 1, that is, including immediately adjacent time points.

^d^Results for the two-fold FCS with a time window width of 2, that is, including two adjacent time points.

Table S4. Performance of various methods for handling 25% missingness in BMI for age z-scores; true OR^a^=1.5(log(OR)=0.4)

| Performance Measure | Method | | | | |
| --- | --- | --- | --- | --- | --- |
|  | Complete Case Analysis | FCS | MVNI | two-fold FCS (width=1)^c^ | two-fold FCS (width=2)^d^ |
| **MCAR** |  |  |  |  |  |
| Absolute Bias^b^ | 0.001 | 0.001 | 0.001 | 0.003 | 0.001 |
| Relative Bias (%) | 0.19 | 0.13 | 0.13 | 0.67 | 0.24 |
| Empirical SE^b^ | 0.017 | 0.017 | 0.017 | 0.017 | 0.017 |
| Model-based SE^b^ | 0.017 | 0.017 | 0.017 | 0.017 | 0.017 |
| Coverage (%) | 95.6 | 95.3 | 95.2 | 94.8 | 95.2 |
| RMSE | 0.017 | 0.017 | 0.017 | 0.017 | 0.017 |
| **MAR (weak)** |  |  |  |  |  |
| Absolute Bias^b^ | 0.007 | 0.000 | 0.000 | 0.002 | 0.001 |
| Relative Bias (%) | 1.82 | 0.12 | 0.11 | 0.42 | 0.12 |
| Empirical SE^b^ | 0.017 | 0.017 | 0.017 | 0.017 | 0.017 |
| Model-based SE^b^ | 0.017 | 0.017 | 0.017 | 0.017 | 0.017 |
| Coverage (%) | 92.2 | 94.4 | 94.7 | 94.1 | 94.9 |
| RMSE | 0.019 | 0.017 | 0.017 | 0.017 | 0.017 |
| **MAR (strong)** |  |  |  |  |  |
| Absolute Bias^b^ | 0.010 | 0.000 | 0.000 | 0.000 | 0.001 |
| Relative Bias (%) | 2.48 | 0.08 | 0.07 | 0.00 | 0.17 |
| Empirical SE^b^ | 0.017 | 0.017 | 0.017 | 0.017 | 0.017 |
| Model-based SE^b^ | 0.017 | 0.017 | 0.017 | 0.017 | 0.017 |
| Coverage (%) | 91.7 | 95.8 | 95.6 | 95.7 | 95.3 |
| RMSE | 0.020 | 0.017 | 0.017 | 0.017 | 0.016 |

Abbreviations: Empirical SE, empirical standard error; FCS, fully conditional specification; MAR, missing at random; MCAR, missing completely at random; Model-based SE, model based standard error; MVNI, multivariate normal imputation; RMSE, root mean square error; two-fold FCS, two-fold fully conditional specification algorithm.

^a^True OR represents the true odds ratio between sleep problems and BMI for age z-scores.

^b^Monte Carlo standard error did not exceed 0.0006.

^c^Results for the two-fold FCS with a time window width of 1, that is, including immediately adjacent time points.

^d^Results for the two-fold FCS with a time window width of 2, that is, including two adjacent time points.

Table S5. Performance of various methods for handling 50% missingness in BMI for age z-scores; true OR^a^=1.5(log(OR)=0.4)

| Performance Measure | Method | | | | |
| --- | --- | --- | --- | --- | --- |
|  | Complete Case Analysis | FCS | MVNI | two-fold FCS (width=1)^c^ | two-fold FCS (width=2)^d^ |
| **MCAR** |  |  |  |  |  |
| Absolute Bias^b^ | 0.001 | 0.000 | 0.000 | 0.004 | 0.001 |
| Relative Bias (%) | 0.24 | 0.03 | 0.03 | 1.08 | 0.33 |
| Empirical SE^b^ | 0.018 | 0.018 | 0.018 | 0.018 | 0.018 |
| Model-based SE^b^ | 0.019 | 0.018 | 0.018 | 0.018 | 0.018 |
| Coverage (%) | 96.4 | 95.3 | 95.4 | 94.3 | 94.7 |
| RMSE | 0.018 | 0.018 | 0.018 | 0.019 | 0.018 |
| **MAR (weak)** |  |  |  |  |  |
| Absolute Bias^b^ | 0.016 | 0.000 | 0.000 | 0.003 | 0.000 |
| Relative Bias (%) | 4.06 | 0.10 | 0.09 | 0.69 | 0.09 |
| Empirical SE^b^ | 0.019 | 0.018 | 0.018 | 0.018 | 0.018 |
| Model-based SE^b^ | 0.019 | 0.018 | 0.018 | 0.018 | 0.018 |
| Coverage (%) | 86.5 | 95.1 | 94.6 | 94.2 | 94.6 |
| RMSE | 0.025 | 0.018 | 0.018 | 0.019 | 0.018 |
| **MAR (strong)** |  |  |  |  |  |
| Absolute Bias^b^ | 0.023 | 0.000 | 0.000 | 0.001 | 0.002 |
| Relative Bias (%) | 5.64 | 0.02 | 0.02 | 0.32 | 0.56 |
| Empirical SE^b^ | 0.019 | 0.018 | 0.018 | 0.018 | 0.018 |
| Model-based SE^b^ | 0.018 | 0.018 | 0.018 | 0.018 | 0.018 |
| Coverage (%) | 76.0 | 95.5 | 96.0 | 95.5 | 95.3 |
| RMSE | 0.029 | 0.018 | 0.018 | 0.018 | 0.018 |

Abbreviations: Empirical SE, empirical standard error; FCS, fully conditional specification; MAR, missing at random; MCAR, missing completely at random; Model-based SE, model based standard error; MVNI, multivariate normal imputation; RMSE, root mean square error; two-fold FCS, two-fold fully conditional specification algorithm.

^a^True OR represents the true odds ratio between sleep problems and BMI for age z-scores.

^b^Monte Carlo standard error did not exceed 0.0006.

^c^Results for the two-fold FCS with a time window width of 1, that is, including immediately adjacent time points.

^d^Results for the two-fold FCS with a time window width of 2, that is, including two adjacent time points.


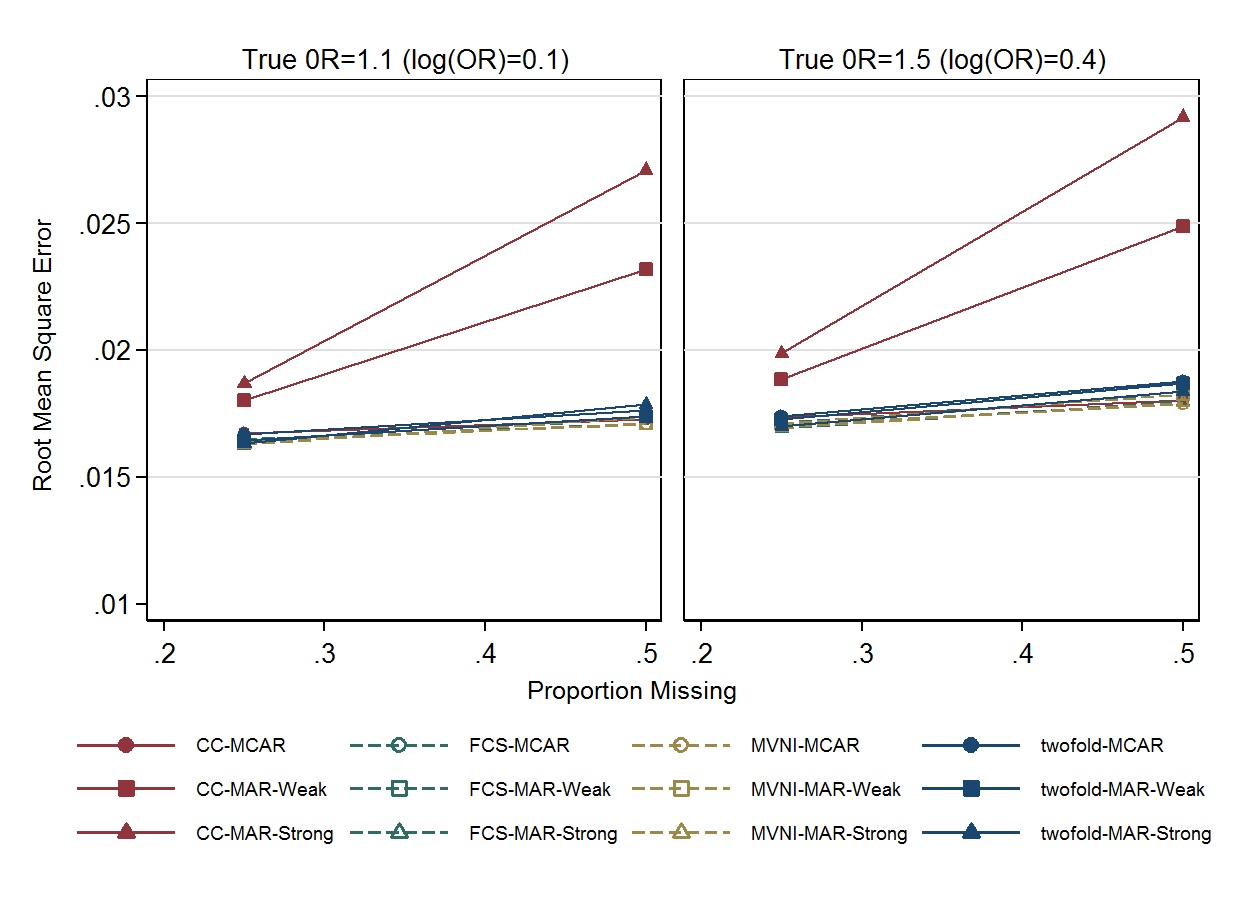
Figure S2. Root mean square error for complete case analysis (CC), fully conditional specification (FCS), multivariate normal imputation (MVNI), and two-fold fully conditional specification (two-fold FCS) for increasing proportions of missing data (0.25, 0.5) under three missing data scenarios and two simulation scenarios; true OR represents the true odds ratio between sleep problems and BMI for age z-scores.


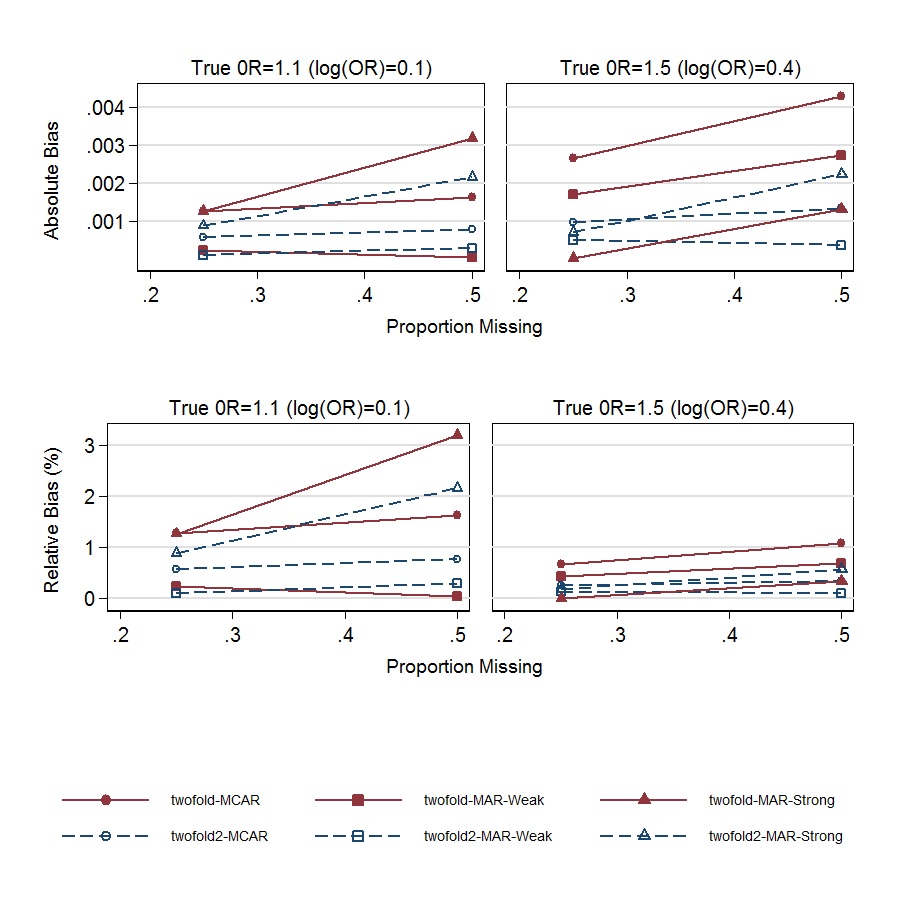
Figure S3. Absolute bias and Relative bias (%) for two-fold fully conditional specification (two-fold FCS) algorithm with time window width 1 and time window width 2 (twofold2) for increasing proportions of missing data (0.25, 0.5) under three missing data scenarios and two simulation scenarios; true OR represents the true odds ratio between sleep problems and BMI for age z-scores.

^a^ Relative bias is calculated as absolute bias relative to the value of the true parameter. As the value of the true parameter (log(OR)) increases from 0.1 to 0.4 in the second simulation scenario, the magnitude of the relative bias drops even though the absolute bias shows a slight increase.


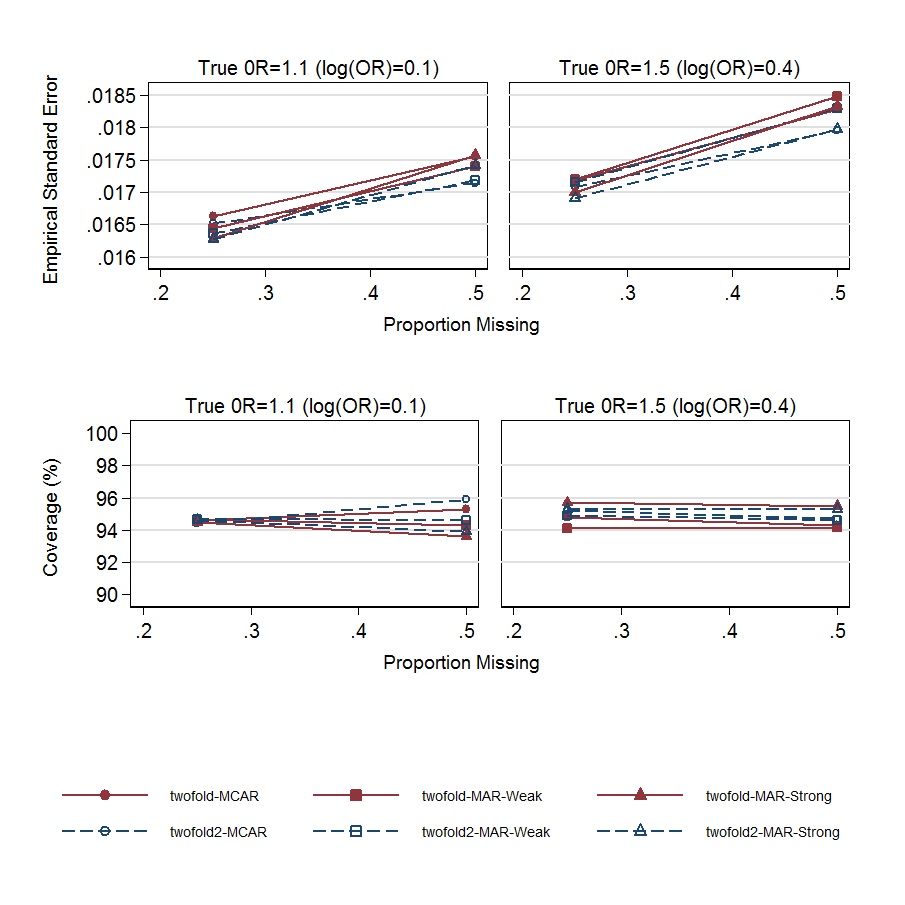
Figure S4. Empirical standard error and Coverage (%) for two-fold fully conditional specification (two-fold FCS) algorithm with time window width 1 and time window width 2 (twofold2) for increasing proportions of missing data (0.25, 0.5) under three missing data scenarios and two simulation scenarios; true OR represents the true odds ratio between sleep problems and BMI for age z-scores.
